# Distinctive features of *Zaprionus indianus* hemocyte differentiation and function revealed by transcriptomic analysis

**DOI:** 10.3389/fimmu.2023.1322381

**Published:** 2023-12-21

**Authors:** Gyöngyi Cinege, Lilla B. Magyar, Henrietta Kovács, Viktória Varga, László Bodai, Nóra Zsindely, Gábor Nagy, Zoltán Hegedűs, Dan Hultmark, István Andó

**Affiliations:** ^1^ Innate Immunity Group, Institute of Genetics, HUN-REN Biological Research Centre, Szeged, Hungary; ^2^ Doctoral School of Biology, University of Szeged, Szeged, Hungary; ^3^ Department of Biochemistry and Molecular Biology, University of Szeged, Szeged, Hungary; ^4^ Laboratory of Bioinformatics, HUN-REN Biological Research Centre, Szeged, Hungary; ^5^ Department of Biochemistry and Medical Chemistry, Medical School, University of Pécs, Pécs, Hungary; ^6^ Department of Molecular Biology, Umea University, Umea, Sweden

**Keywords:** hemocyte, multinucleated giant hemocyte, immune response, *Drosophila*, wound healing, invasive, transcriptome, *Zaprionus indianus*

## Abstract

**Background:**

Insects have specialized cell types that participate in the elimination of parasites, for instance, the lamellocytes of the broadly studied species *Drosophila melanogaster*. Other drosophilids, such as *Drosophila ananassae* and the invasive *Zaprionus indianus*, have multinucleated giant hemocytes, a syncytium of blood cells that participate in the encapsulation of the eggs or larvae of parasitoid wasps. These cells can be formed by the fusion of hemocytes in circulation or originate from the lymph gland. Their ultrastructure highly resembles that of the mammalian megakaryocytes.

**Methods:**

Morphological, protein expressional, and functional features of blood cells were revealed using epifluorescence and confocal microscopy. The respective hemocyte subpopulations were identified using monoclonal antibodies in indirect immunofluorescence assays. Fluorescein isothiocyanate (FITC)-labeled *Escherichia coli* bacteria were used in phagocytosis tests. Gene expression analysis was performed following mRNA sequencing of blood cells.

**Results:**

*D. ananassae* and *Z. indianus* encapsulate foreign particles with the involvement of multinucleated giant hemocytes and mount a highly efficient immune response against parasitoid wasps. Morphological, protein expressional, and functional assays of *Z. indianus* blood cells suggested that these cells could be derived from large plasmatocytes, a unique cell type developing specifically after parasitoid wasp infection. Transcriptomic analysis of blood cells, isolated from naïve and wasp-infected *Z. indianus* larvae, revealed several differentially expressed genes involved in signal transduction, cell movements, encapsulation of foreign targets, energy production, and melanization, suggesting their role in the anti-parasitoid response. A large number of genes that encode proteins associated with coagulation and wound healing, such as phenoloxidase activity factor-like proteins, fibrinogen-related proteins, lectins, and proteins involved in the differentiation and function of platelets, were constitutively expressed. The remarkable ultrastructural similarities between giant hemocytes and mammalian megakaryocytes, and presence of platelets, and giant cell-derived anucleated fragments at wound sites hint at the involvement of this cell subpopulation in wound healing processes, in addition to participation in the encapsulation reaction.

**Conclusion:**

Our observations provide insights into the broad repertoire of blood cell functions required for efficient defense reactions to maintain the homeostasis of the organism. The analysis of the differentiation and function of multinucleated giant hemocytes gives an insight into the diversification of the immune mechanisms.

## 1 Introduction

Over the course of evolution, mechanisms of the innate immune system have diversified into a broad repertoire of various effector cells, which participate in the production of antimicrobial peptides, phagocytosis of microbes, encapsulation of large particles, blood clotting, wound healing, regeneration, and tissue remodeling. As the transcriptional and epigenetic regulators and the signal transduction pathways are all phylogenetically conserved between insects and vertebrates (1, 2), *Drosophila* has become a well-established model organism for the analysis of cellular innate immune reactions and host–pathogen interactions.

In *Drosophila melanogaster*, four main classes of effector blood cells, called hemocytes, were described: the phagocytic plasmatocytes, the melanizing crystal cells, the encapsulating lamellocytes, and the recently discovered primocytes (PSC-like cells) (3–9). The lamellocytes attach to and spread around large particles, including eggs and larvae of parasitoids, and form a multilayered capsule to block parasitoid development. Genetic variation among fly species for resistance against invaders, and variation in virulence of wasp species against the flies, will determine the effectiveness and success of the defense against parasitoids. We have previously found that several fly species, such as those of the *ananassae* subgroup and *Zaprionus indianus* of the *vittiger* subgroup of Drosophilidae, produce a new encapsulating hemocyte type, a syncytium of blood cells: the highly motile multinucleated giant hemocyte (MGH) (10–12).

Multinucleation is a form of endomitosis, where the completion of mitotic events through nuclear division in the absence of cytokinesis leads to multinucleated cells. Generally, most cells maintain a diploid state, but when there is a demand for high rates of metabolic activity and biosynthesis, amplification of the genome provides an advantage. In many cases, multinucleated cells are of hematopoietic origin (13, 14), and their presence is often associated with infections (15–17). Moreover, multinucleated epidermal cells have been detected around wounds in *Drosophila* larvae, which eliminated intracellular spaces and helped seal the wound site (18). It is clear that multinucleation results in an increase of genetic material, which permits these cells to react quickly and robustly, contributing to an efficient effector function.

Combined immuno-electron microscopic and transcriptomic analysis of *Drosophila ananassae* MGHs revealed that these cells possess a characteristic cellular ultrastructure, which is highly supported by their gene expression profile (12), ensuring efficient defense. Similarly, in the cytoplasm of the *Z. indianus* MGHs, we detected a large number of canals and sinuses that communicate with the extracellular space (11), providing a substantial contact surface between the cell and the environment, which is a considerable metabolic advantage.

We previously showed that MGHs of *Z. indianus* belong to a cell type named giant hemocytes, defined by staining with the 4G7 antibody. In addition to the multinucleated cells, the 4G7-positive cell fraction also comprised elongated, single-nucleated blood cells, named nematocytes (19), and anucleated cell fragments surrounded by the plasma membrane, released from the previous two (11). Giant hemocytes and their anucleated cytoplasmic derivatives are also present in naïve larvae (11). We observed that MGHs of *D. ananassae* and *Z. indianus* formed *via* cell fusion in the circulation (10, 11); however, the hematopoietic organ, the lymph gland of *Z. indianus* also contributed to the formation of these cells (11).

In this study, we aimed to obtain deeper insights into the differentiation and function of the MGHs. We show that *D. ananassae* and *Z. indianus*, species developing MGHs, are highly resistant to parasitoid wasps. Furthermore, in *Z. indianus*, we identified a novel multinucleated cell type, the spherical “large plasmatocyte”, developing specifically after parasitoid wasp infection and representing a new source for differentiation of giant hemocytes responsible for encapsulation of the invader. Transcriptomic analysis revealed differential expression of several genes in the hemocytes of parasitoid wasp-infected samples, which encode for proteins involved in biological processes required in anti-parasitoid defense reactions. In a comparative analysis of *D. melanogaster*, *D. ananassae*, and *Z. indianus* transcriptomes, we present several sets of genes that are responsible for the characteristic features of the highly effective defense against parasitoids and highlight the involvement of an ancient mechanism with the contribution of MGHs.

Moreover, here, we found that the 4G7-positive anucleated fragments released from the giant hemocytes accumulate at the wound sites of both naïve and wasp-induced animals. In line with this, we identified several genes expressed in both uninduced and induced blood cell samples, which possess orthologs of genes involved in the wound healing processes of *D. melanogaster* and mammalian species.

The analysis of MGHs in the highly resistant fruit fly species gives insight into the diversification of the immune mechanisms, which likely have provided evolutionary advantages in a broad range of organisms that develop MGHs.

## 2 Materials and methods

### 2.1 Insect stocks and culturing

*D. ananassae* wild type (14024-0371.13) was obtained from the UC San Diego *Drosophila* stock center. *Z. indianus* wild-type strain #3 (19) was kindly provided by Bálint Z. Kacsoh (University of Pennsylvania, USA). *D. melanogaster* wild type (Oregon-R) was ordered from Bloomington Drosophila Stock Center. Each strain was kept at 25°C on standard yeast-cornmeal food. The *Leptopilina heterotoma* (14) and *Leptopilina victoriae* (UNK) wasp strains were kindly provided by Prof. Todd Schlenke (University of Arizona, USA). Wasps were maintained on *D. melanogaster* Oregon-R.

### 2.2 Parasitization assay

For infection with the parasitic wasps, 60 early second instar larvae were placed together on vials containing standard yeast-cornmeal food with 15 female wasps for 6 hours at 25°C. For eclosion experiments, 48 h after the wasp infection, 10 larvae were dissected from each vial and scored for the presence of the parasitoid eggs or larvae. If each of the 10 tested larvae carried parasitoids, we considered the rest of the samples from the respective vial 100% infected. Following pupariation, the number of eclosed fly and wasp adults was counted, and their proportion was determined. The results of four independent experiments are shown.

### 2.3 Injury and microbead injection

Early second instar larvae were washed in *Drosophila* Ringer solution and one by one placed on a sterile Petri dish for the respective treatment, which was carried out on the dorsal part of the fifth segment, using sterile, 100-μm-diameter minutien pins or a glass capillary. For septic injury, before treatment, the sterile minutien pins were dipped in a 1:1 mixture of concentrated overnight grown Gram-positive (*Bacillus subtilis*, SzMC 0209) and Gram-negative (*Escherichia coli*, SzMC 0582) bacterial suspension (both bacteria obtained from the Szeged Microbial Collection, University of Szeged, Hungary) or a 50% concentrated suspension of *Beauveria bassiana* entomopathogenic fungus spores (Kwizda Agro, Artis Pro) in sterile phosphate-buffered saline (PBS). For injection, a volume of 0.1 μl of 15-μm-diameter FluoSpheres polystyrene microbeads (Invitrogen, Carlsbad, CA, USA) in sterile *Drosophila* Ringer solution was injected into the larvae at the dorsal part of the fifth segment. A control sterile *Drosophila* Ringer solution was used. After injury or injection, the larvae were carefully transferred into a vial with standard yeast-cornmeal food and used after 48 h for blood cell differentiation assays. For wound healing experiments, early third instar naïve or *L. victoriae*-infected larvae were wounded using a sterile minutien needle as described above, and after a 2-h incubation time, cuticle samples were prepared and immunostaining was carried out.

### 2.4 Video microscopy

Seventy-two hours after *L. victoriae* wasp infection, larvae were dissected in Complete Schneider’s medium (CSM): Schneider’s medium (Lonza, Basel, Switzerland) supplemented with 5% fetal bovine serum (GIBCO, Grand Island, NY, USA) and 0.01% 1-phenyl-2-thiourea (Sigma, Darmstadt, Germany). The live hemocytes were analyzed using an Alpha XDS-1T inverted microscope at room temperature. Photographs were taken using a Nikon D5300 DSLR camera. Shooting duration was 75 min with 13-s intervals (**Supplementary Movie 1**). The images were edited using the Adobe Lightroom CC 2015 program, and the movie was made using FIJI (https://fiji.sc/). Olympus Fluoview and Olympus 3D viewer software were used to prepare the 3D animation of the 4G7 and DAPI-stained wound site.

### 2.5 Preparation of hemocyte and cuticle samples

For hemocyte samples, larvae were dissected at respective time points in CSM on glass microscope slides. Blood cells were adhered to the glass surface for 1 h, followed by fixation with acetone for 6 min, air-dried, and blocked with 0.1% bovine serum albumin (BSA) in PBS for 20 min. For cuticle preparations, larvae were opened in CSM along the longitudinal axis, and the digestive tracts and fat bodies were removed. The carcasses were then fixed in 2% paraformaldehyde for 10 min, washed three times for 5 min in PBS, and blocked with 0.1% BSA in PBS supplemented with 0.01% Triton X-100 for 20 min. Samples were incubated with the antibodies as described below.

### 2.6 Antibodies and indirect immunofluorescence

After blocking, hemocytes and cuticle preparations were incubated with the primary antibodies for 1 h at room temperature. The 5C3 antibody reacted with spherical cells of different sizes, the plasmatocytes, the 4G7 specific for the giant hemocytes, a subpopulation of cells including bi- or multinucleated giant hemocytes, nematocytes (elongated blood cells with a single nucleus), and filariform anucleated structures (11). The anti-human CD45, T2/48 antibody (20) was used as the negative control. The monoclonal antibodies were generally used as undiluted hybridoma supernatants. Following incubation with the primary antibodies, samples were washed three times for 5 min in PBS and incubated with the secondary antibodies for 45 min. As a secondary antibody, the anti-mouse Alexa Fluor 488 goat antibody (Invitrogen, 1:1,000 dilution) was used. For double staining, a 1:1 mixture of the anti-mouse IgM Cross-Adsorbed fluorescein isothiocyanate (FITC) (P.A.R.I.S. 1:1,000 dilution) and anti-mouse IgG2a Cross-Adsorbed Alexa Fluor 568 (Invitrogen, 1:1,000 dilution) secondary antibodies were used. To visualize the nuclei, DAPI (Sigma) at a concentration of 2.5 µg/ml was added to the secondary antibodies. After incubation with the secondary antibodies, samples were washed three times for 5 min and covered with Fluoromount-G medium and a coverslip. Samples were analyzed using a Zeiss Axioscope 2MOT epifluorescence or an Olympus FV1000 confocal microscope.

### 2.7 Cell measurements, detection of the large plasmatocytes, and statistics

For each sample, hemocyte images of two randomly selected areas were acquired using the 20× objective of a Zeiss Axioscope 2MOT epifluorescence microscope. Cell measurements and hemocyte counts were determined with the ImageJ program (http://imagej.nih.gov/ij/) using microscopic images with standard size, resolution, and magnification. The cell areas were normalized to that of naïve plasmatocytes; therefore, the average area of plasmatocytes was set to 1. As the naïve plasmatocytes possessed lower relative areas than 5 (5 times the average size of naïve plasmatocytes), 5C3-positive cells, which exhibited larger areas, were considered to be large plasmatocytes. Images captured in three independent experiments were analyzed using at least 30 larvae in each. A relative area of 2,000 randomly selected cells is shown. The significance of the differences was determined by Student’s *t*, one-way ANOVA, and Tukey’s honestly significant difference (HSD) tests.

### 2.8 *In vivo* phagocytosis assay

The phagocytosis assay was carried out on *L. victoriae* parasitoid wasp-induced *Z. indianus* larvae as described previously (11). The results of three independent experiments, with 24 larvae in each, were combined.

### 2.9 Collection of samples for sequencing library preparation, RNA sequencing, and bioinformatic analysis of transcriptome data

For transcriptomic analysis, blood cells from 100 age-matched uninduced and 100 *L. victoriae*-induced (72 hours after the parasitoid wasp infection) *Z. indianus* larvae were harvested, and RNA was isolated using an RNeasy mini kit (Qiagen, Valencia, CA, USA). Three parallel samples were used for each group. The quantity and integrity of total RNA samples were determined by capillary gel electrophoresis using a 2100 Bioanalyzer instrument (Agilent, Santa Clara, CA, USA) using Agilent RNA 6000 Nano Kit. For RNA sequencing, mRNA was isolated from 220 ng total RNA per sample using a NEBNext Poly(A) mRNA Magnetic Isolation Module (New England Biolabs (NEB), Ipswich, MA, USA); then, strand-specific sequencing libraries were generated using a NEBNext Ultra II Directional RNA Library Prep Kit for Illumina (NEB) with NEBNext Multiplex Oligos for Illumina (NEB) following the protocol of the manufacturer. Indexed sequencing libraries were validated and quantitated using an Agilent DNA 1000 kit in a 2100 Bioanalyzer instrument; then, libraries were pooled in equimolar ratios. After denaturing, the library pool was diluted to 15-pM concentration and sequenced in a MiSeq DNA sequencer (Illumina, San Diego, CA, USA) using a MiSeq Reagent Kit v3 (150-cycle) producing 2 × 75 bp paired-end reads. Base calling and generation of FASTQ sequence files were performed using BaseSpace Sequence Hub (https://basespace.illumina.com) algorithms. FASTQ files were quality trimmed using the TrimGalore software and then aligned to the *Z. indianus* reference genome (21) using HISAT2. To determine the number of sequence reads mapped to each gene, the reference transcriptome was imported to the R software using the GenomicFeatures package, and then read counts were calculated using the GenomicAlignments R package. DESeq2 analysis was carried out for data normalization and differential gene expression analysis. Genes with a read count <10 were filtered out from the analysis. Only those genes were considered significantly differentially expressed in uninduced and *L. victoriae*-induced *Z. indianus* blood cells, where the Benjamini–Hochberg adjusted p-value was <0.05 and the absolute log_2_FoldChange was ≥1. A gene was considered expressed in a given sample if the Fragments per kilobase of exon model per million reads mapped (FPKM) values were >1. Furthermore, a protein was considered ortholog when the coverage was at least 20%, the identity was higher than 40%, and the E-value representing the quality of the alignment was lower than 0.0001. The National Center for Biotechnology Information (NCBI) database was used to find ortholog sequences. Functional data regarding *D. melanogaster* orthologs were acquired using Flybase, those of mammalian orthologs were acquired using Ingenuity Pathways Analysis (IPA), and other gene information was collected from the http://datadryad.org/stash/dataset/doi:10.5061/dryad.866t1g1n3 database. Gene ontology (GO) enrichment analysis for the differentially expressed genes was carried out with the software R, using the clusterProfiler package: https://bioconductor.org/packages/devel/bioc/vignettes/clusterProfiler/inst/doc/clusterProfiler.html. As there is no information available concerning the *Z. indianus* genes and proteins, their *D. melanogaster* orthologs were used.

## 3 Results

### 3.1 Species developing MGHs are highly resistant to parasitoids

**Figure 1:**
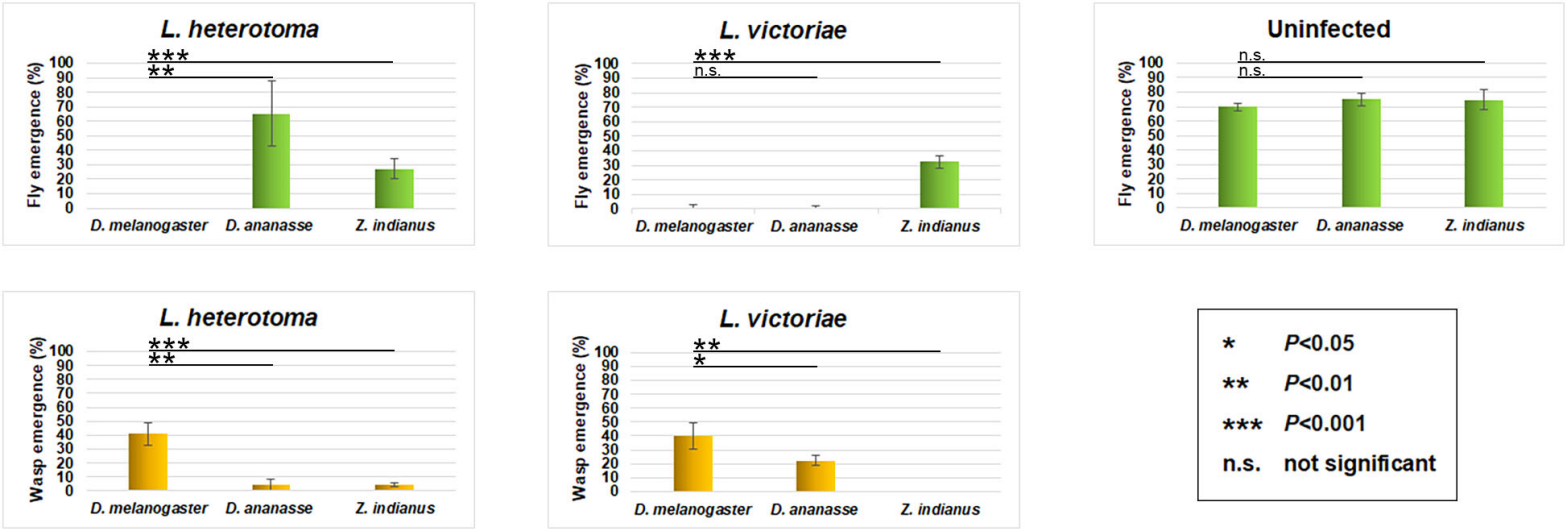
Eclosion success of two generalist wasp species. *Drosophila ananassae*, *Zaprionus indianus*, and *Drosophila melanogaster* larvae were infected with *Leptopilina heterotoma* and *Leptopilina victoriae* parasitoid wasps. The data of four independent experiments were cropped, with 50 larvae in each. The error bars indicate the standard deviation. Student’s *t*-test was used for statistical analysis.

The fly and wasp eclosion rates of parasitized *D. ananassae*, *Z. indianus*, and *D. melanogaster* were compared (**Figure 1**). Infections were carried out using two generalist parasitoid wasp species, *L. heterotoma* and *L. victoriae*. The proportional outcome of wasp-attacked flies was analyzed. We observed that both wasp species eclosed at a rate of approximately 40% from *D. melanogaster*, but they eclosed at a significantly lower rate from *D. ananassae* and *Z. indianus*; meanwhile, the fly survival rate of these species was in general higher than in *D. melanogaster* (**Figure 1**).

### 3.2 Parasitoid wasp infection induces differentiation of large plasmatocytes

**Figure 2:**
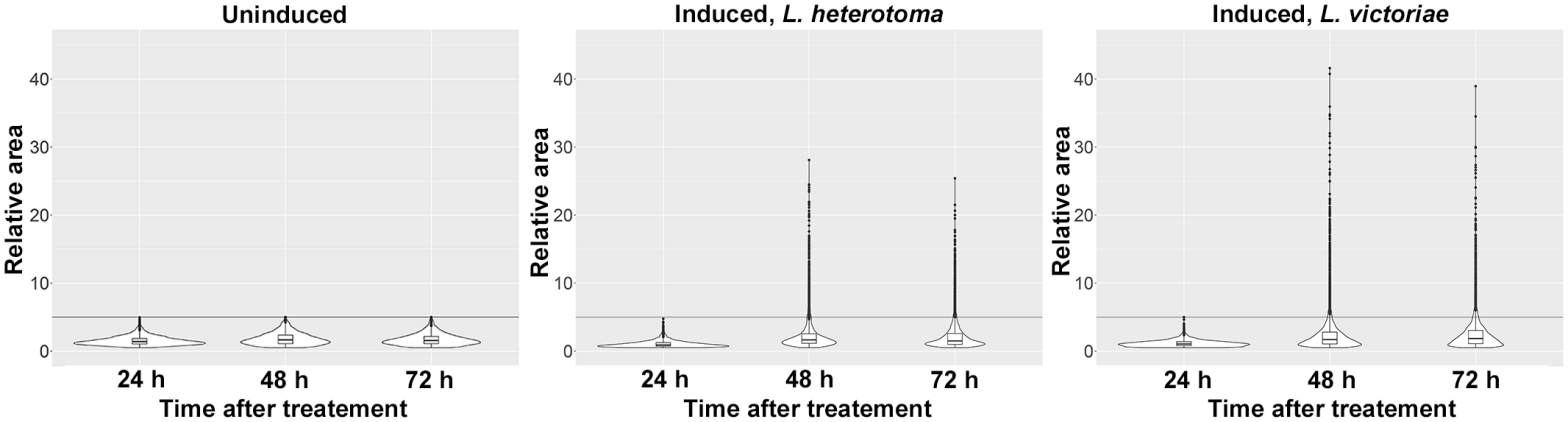
Time-lapse analysis of large plasmatocyte differentiation following parasitoid wasp infection. *Leptopilina heterotoma* and *Leptopilina victoriae* parasitoids were used to infect early second instar *Zaprionus indianus* larvae. Blood cells were harvested 24 h, 48 h, and 72 h after infection. As controls, age-matched uninduced larvae were used. Violin plots were generated in RStudio to interpret data distribution. For each plot, relative area of 2,000 randomly selected 5C3-positive cells was applied. The first quartile, the median, and the third quartile are shown.

We previously observed that multinucleation in *Z. indianus* was not restricted to MGHs, as a novel cell type appeared after parasitic infection: a class of plasmatocytes, spherical large cells, hereafter called large plasmatocytes, which occasionally had numerous nuclei. To reveal the dynamics of the appearance of large plasmatocytes, we carried out a time-lapse analysis. We infected *Z. indianus* larvae with *L. heterotoma* and *L. victoriae*; we isolated blood cells 24 h, 48 h, and 72 h after the wasp infection and stained them with the plasmatocyte-specific 5C3 antibody (11); and we determined the relative area of the 5C3-positive hemocytes. We detected the large plasmatocytes 48 h and 72 h after infection, and the relative area of these cells was significantly higher at 48 h compared to 72 h after *L. heterotoma* parasitization (**Figure 2**). By comparison, the size of the plasmatocytes of age-matched uninduced animals remained constant.

**Figure 3:**
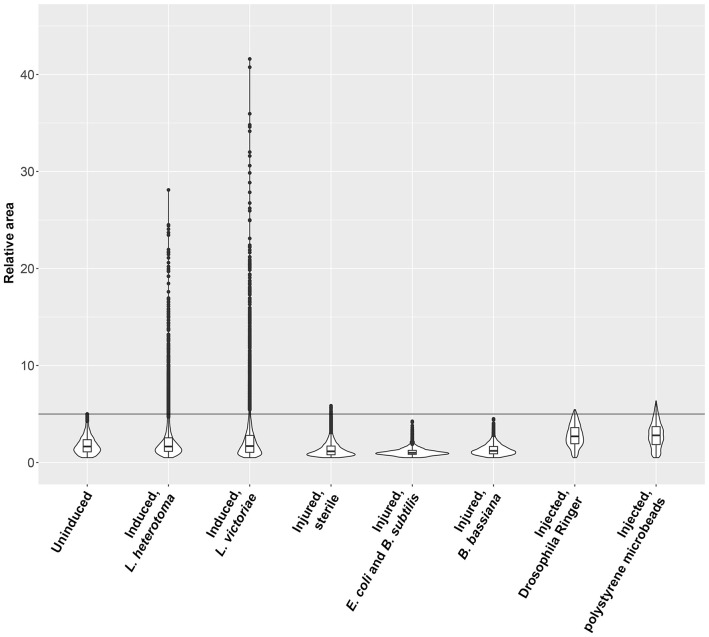
Parasitic wasp infection induces differentiation of large plasmatocytes. The respective treatments were carried out on second instar *Zaprionus indianus* larvae, and 5C3-positive blood cells were analyzed after 48 h. Naïve, sterile wounded, and *Drosophila* Ringer-injected larvae were used as controls. Violin plots were generated in RStudio using relative area of 2,000 randomly selected cells for each plot. The first quartile, the median, and the third quartile are shown.

Next, we tested whether other immune stimuli, in addition to the parasitoid wasp attacks, could trigger the formation of large plasmatocytes. We used suspensions of *B. subtilis* (Gram-positive), *E. coli* (Gram-negative) bacteria, and an entomopathogenic fungus *B. bassiana* for infection. We applied sterile wounding as the control. Furthermore, we tested whether the presence of large foreign particles could stimulate the development of this cell type. Hence, we injected inert microbeads in sterile *Drosophila* Ringer solution into the hemocoel of second instar *Z. indianus* larvae. We injected sterile *Drosophila* Ringer solution as the control. We carried out the analysis 48 h after the respective treatments, which was the peak of large plasmatocyte differentiation after parasitoid wasp infection. We found that septic injury, sterile wounding, or injection of microbeads did not induce differentiation of large plasmatocytes (**Figure 3**). Although the injection of *Drosophila* Ringer, with or without microbeads, triggered a slightly increased relative area of plasmatocytes, we concluded that the appearance of large plasmatocytes is specifically connected to parasitic wasp infection (**Figure 3**).

### 3.3 Large plasmatocytes display a binary immune phenotype

**Figure 4:**
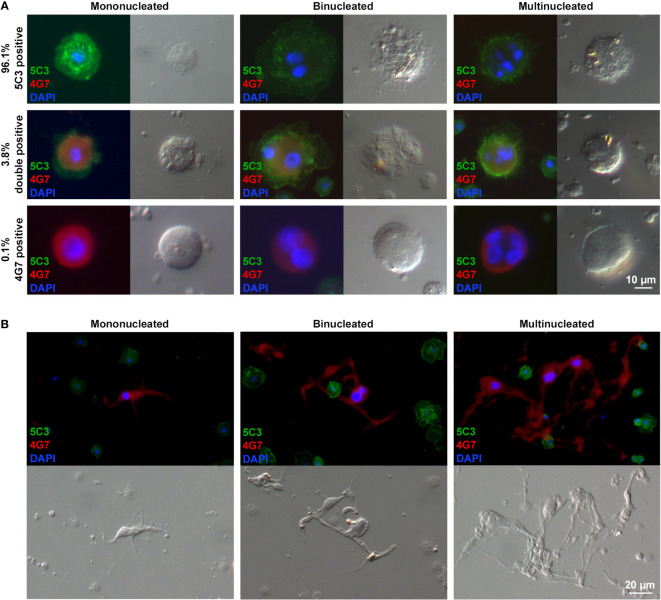
Certain large plasmatocytes display binary immune phenotypes or express the giant cell-specific antigen. Forty-eight hours after *Leptopilina victoriae* infection, hemocytes were double stained, using the plasmatocyte-specific 5C3 (IgM), the giant cell-specific 4G7 (IgG2a) monoclonal antibodies, and the respective isotype-specific secondary antibodies. **(A)** The giant cell-specific 4G7 antigen was presented by 3.9% of the large plasmatocytes. Mono-, bi-, and multinucleation were detected in each cell type. The large plasmatocytes carrying exclusively the 4G7 antigen possessed enlarged nuclei **(B)**. The giant cells 72 h after wasp infection carried one, two, or more enlarged nuclei. Detection was performed using an epifluorescence microscope (Zeiss Axioscope 2 MOT).

The phenotypic analysis of large plasmatocytes revealed that a fraction of these cells expressed the 4G7 giant cell-specific antigen. As mentioned before, the giant cell fraction of *Z. indianus* includes MGHs, elongated cells with a single nucleus (nematocytes), and anucleated cytoplasmic fragments derived from the previous two, all involved in the encapsulation of parasitoids (11, 19). Expression of the 4G7 antigen on certain large plasmatocytes is suggestive of a transient state between the large plasmatocytes and giant hemocytes; hence, we applied double staining with the combinations of the 5C3 plasmatocyte-specific antibodies and the 4G7 giant hemocyte-specific antibodies in the same samples to analyze the expression rate of cell type-specific antigens. Hemocytes were isolated 48 h after *L. victoriae* parasitoid wasp infection. Our data showed that while 96.1% of the large plasmatocytes were only 5C3-positive, 0.1% presented the 4G7 antigen exclusively, and 3.8% were double-positive (**Figure 4A**). Each cell category included mononucleated, binucleated, and multinucleated cells. While the size of the nuclei in the 5C3-positive large plasmatocytes varied, the 4G7-positive large plasmatocytes carried exclusively enlarged nuclei (**Figure 4A**), a characteristic feature of the giant hemocytes in the wasp-infected larvae (11) (**Figure 4B**).

**Figure 5:**
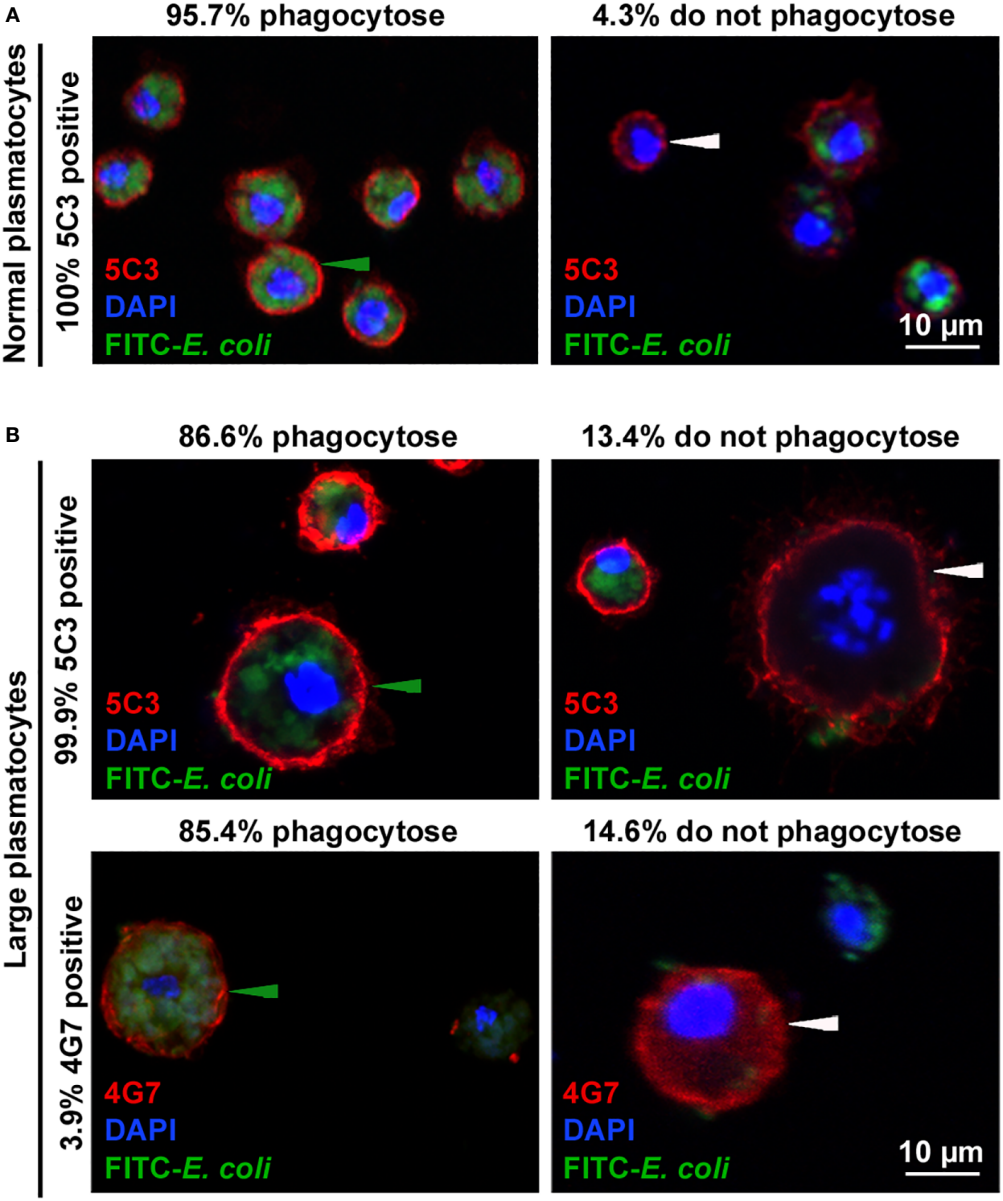
Phagocytic ratio of large plasmatocytes decreased when compared to that of normal plasmatocytes. Fluorescein isothiocyanate (FITC)-labeled *Escherichia coli* bacteria were used in combination with the respective antibodies. Green arrowheads point to the plasmatocytes that phagocytosed *E coli*, and white arrowheads show those that did not. Analysis was performed using an Olympus FV1000 confocal LSM microscope, and images in the nucleus plane were used. Results of three independent experiments were considered, each with 24 larvae. **(A)** High ratio of the normal plasmatocytes engulfed bacteria. **(B)** Lower ratio of large plasmatocytes with both immunological phenotypes possessed phagocytic capacity.

Plasmatocytes engulf bacteria, but encapsulating giant hemocytes are not phagocytic (11), so we compared the phagocytic capacity of the normal-sized and the large plasmatocytes in relation to their antigen expression phenotypes. We infected second instar larvae with *L. victoriae* parasitoids, and 48 h after wasp infection, we injected larvae with FITC-labeled *E. coli* bacteria. One hour after the injection of bacteria, we isolated and subjected hemocytes to indirect immunofluorescence analysis using the 5C3 and 4G7 discriminative antibodies. We found that 4.3% of the 5C3-positive normal-sized plasmatocytes did not engulf bacteria (**Figure 5A**), while 13.4% of the 5C3-positive large plasmatocytes and 14.6% of the 4G7-positive large plasmatocytes did not take up bacteria (**Figure 5B**). Although there was no considerable difference between the phagocytic capacities of 5C3- and 4G7-positive large plasmatocytes, this finding shows that large plasmatocytes are less phagocytic than normal-sized plasmatocytes.

Video microscopic analysis of *L. victoriae*-infected larval hemocytes revealed that large spherical cells changed shape and became elongated cells, morphologically corresponding to the giant hemocytes (**Supplementary Movie 1**). This finding indicates the phenotypic plasticity of the large plasmatocytes differentiating after parasitoid wasp infection.

### 3.4 Anucleated fragments accumulate at wound sites

**Figure 6:**
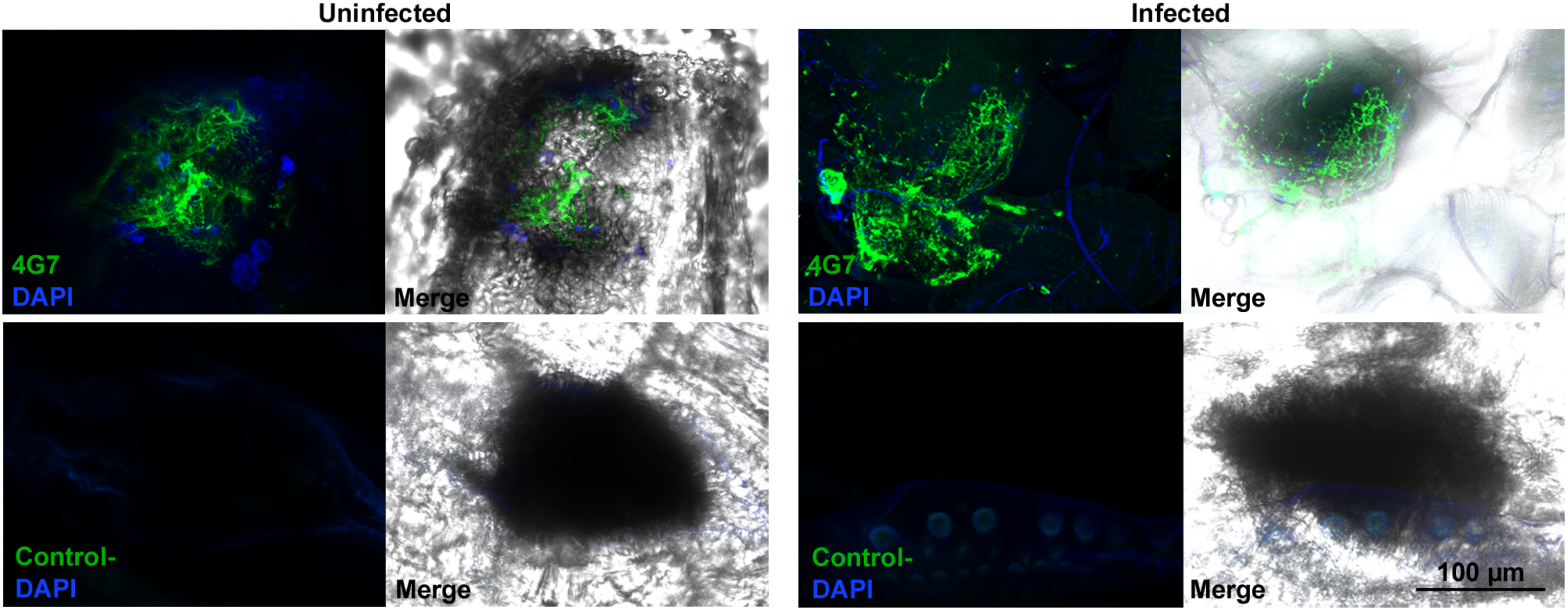
Anucleated 4G7-positive fragments accumulated at the wound sites. Cuticle preparations were performed 2 h after wounding of naïve and wasp-induced larvae and stained with the 4G7 antibody. As a negative control, the T2/48 monoclonal antibody was used. Detection was performed using an Olympus FV1000 confocal LSM microscope.

We previously found that the giant hemocytes of *Z. indianus*, including MGHs, are constitutively present in the hemolymph, possess a characteristic ultrastructure as they carry an elaborate system of canals and sinuses in their cytoplasm, and release a large number of anucleated cytoplasmic fragments (11). We observed that mammalian megakaryocytes, which release anucleated cytoplasmic fragments, the platelets, involved in blood clotting and wound remediation, share similar ultrastructural characteristics with the encapsulating 4G7-positive giant hemocytes of *Z. indianus* (12) because they possess a highly tortuous, invaginated membrane system (22, 23). Hence, we considered whether, by analogy, the anucleated fragments derived from the giant hemocytes of *Z. indianus* could be involved in wound healing. To test this idea, we wounded early third instar naïve or *L. victoriae*-infected larvae, and 2 h later, we examined the wound sites by indirect immunofluorescence assay using the giant cell-specific 4G7 antigen. We observed that the 4G7-positive fragments accumulated at wound sites of both naïve and *L. victoriae*-infected *Z. indianus* larvae (**Figure 6**). This is also supported by 3D confocal picture reconstruction of a 4G7- and DAPI-stained naïve larval sample (**Supplementary Movie 2**, **Supplementary Figure 1**). Although we detected extensive accumulation of the anucleated fragments at the wound sites, the presence of nucleated giant hemocytes cannot be excluded. We suppose therefore that this cell type fulfills an important, so far unrecognized role in cuticle remediation.

### 3.5 Gene expression patterns of hemocytes underlie their functional characteristics

To gain insights into the molecular mechanisms accountable for effective immune defense reactions and to recognize feasible factors involved in wound healing processes, we analyzed the transcriptomic content of hemocytes isolated from age-matched naïve and *L. victoriae*-infected *Z. indianus* larvae. In total, 7,052 different gene transcripts were detected (**Supplementary Table 1**). As there is no available information on *Z. indianus* proteins, the possible function of the genes expressed in blood cells of this species was generally considered on the basis of their *D. melanogaster* orthologs.

First, to identify genes that could participate in the immune defense of *Z. indianus* against parasitoids, we compared the gene expression profile of naïve and wasp-induced hemocyte samples. After normalization, 648 genes were differentially expressed between the induced and uninduced samples (**Supplementary Table 2**). Of these, 374 and 274 genes were expressed at significantly higher and lower levels, in induced and uninduced blood cells, respectively, including 37 genes that were detected exclusively in induced and 12 genes exclusively in uninduced hemocytes (**Supplementary Table 2**). *D. melanogaster* orthologs for 574 of the differentially expressed genes could be identified. Among these, several encode proteins with a role in signal transduction, cell movement, encapsulation of foreign particles, melanization, anabolism, and elimination of microbes (**Table 1**). GO enrichment for “biological process” revealed high expression of genes whose protein products were mainly involved in innate immune defense reactions, amino acid biosynthesis, actin cytoskeleton organization, and cell–cell junctions (**Supplementary Figure 2**). These enrichments are possibly due to the high energy demand, motility, and fusion of the giant hemocytes, processes required for effective parasitoid encapsulation. Furthermore, GO enrichment analysis in the “cellular component” category revealed that genes overexpressed in hemocytes of parasitoid wasp-infected samples encode for proteins that are mostly localized in the actin cytoskeleton, plasma membrane, and cell junctions (**Supplementary Figure 3**), which in addition suggest the importance of the encapsulation reaction, involving cellular contacts, cytoskeletal rearrangements, and adhesion to the parasitoid. Both the up- and downregulated genes could have essential functions in immune responses mediated by the activated blood cells (**Supplementary Table 2**). No orthologs were identified for 74 of the 648 differentially expressed genes, either because they lacked recognizable *D. melanogaster* homologies or because they belong to expanded and rapidly evolving gene families, such as serine proteases, fibrinogen-related proteins (FREPs), and C-type lectins, with uncertain orthology relationships.

**Table 1:** Predicted function of those *Zaprionus indianus* genes that were up- or downregulated in parasitoid wasp-induced samples and possessed *Drosophila melanogaster* orthologs (listed).

**Signal transduction** ***Upregulated*:** Lgr4 (ZIND16G_00006424), mAChR-C (ZIND16G_00004427), CG7497 (ZIND16G_00008737), Proc-R (ZIND16G_00002261), AdoR (ZIND16G_00000124), Galphaf (ZIND16G_00000435), moody (ZIND16G_00002695), Rgk2 (ZIND16G_00005366), Ir8a (ZIND16G_00007061), CG34393 (ZIND16G_00006027), hh (ZIND16G_00003155), Pvf3 (ZIND16G_00008540), Coop (ZIND16G_00006309), p130CAS (ZIND16G_00006792), fs(1)M3 (ZIND16G_00000600), trio (ZIND16G_00001152), Ac78C (ZIND16G_00006086), RSG7 (ZIND16G_00000004), Pld (ZIND16G_00002971), homer (ZIND16G_00004599), gwl (ZIND16G_00009480), PGRP-SC2 (ZIND16G_00004509), PGRP-LB (ZIND16G_00003503), ImpL2 (ZIND16G_00002042), chrb (ZIND16G_00000283), Mnr (ZIND16G_00002683), pigs (ZIND16G_00003317), ric8a (ZIND16G_00002142), CG10359 (ZIND16G_00000441), I-2 (ZIND16G_00000398), P32 (ZIND16G_00004766), Rab32 (ZIND16G_00008079), tum (ZIND16G_00006586), dlp (ZIND16G_00006167), S6k (ZIND16G_00001109), cGlr1 (ZIND16G_00005284), trbd (ZIND16G_00008915), Rala (ZIND16G_00002638), Cp1 (ZIND16G_00005802) ***Downregulated*:** hec (ZIND16G_00005861), Ant2 (ZIND16G_00004162), Alk (ZIND16G_00007368), CG32944 (ZIND16G_00003953), CG4950 (ZIND16G_00002356), CG9391 (ZIND16G_00006079), REPTOR (ZIND16G_00001021), unc-5 (ZIND16G_00007900), sev (ZIND16G_00003697), PGRP-LC (ZIND16G_00008135), Dok (ZIND16G_00004125), magu (ZIND16G_00006621), Men (ZIND16G_00007840), LKRSDH (ZIND16G_00005353), Mob2 (ZIND16G_00001509), Ptp4E (ZIND16G_00004064), Tab2 (ZIND16G_00007372), Doa (ZIND16G_00004009), aru (ZIND16G_00002198), mthl10 (ZIND16G_00001945), CG46491 (ZIND16G_00007929), Pkc98E (ZIND16G_00000763), Thor (ZIND16G_00005322), melt (ZIND16G_00001114), spz (ZIND16G_00008556), Lar (ZIND16G_00009598), Eip75B (ZIND16G_00002603), Lk6 (ZIND16G_00002815), grh (ZIND16G_00005303), ebd1 (ZIND16G_00000358), foxo (ZIND16G_00008428), Ziz (ZIND16G_00008832), Cirl (ZIND16G_00004761), TP53INP (ZIND16G_00006652), gcl (ZIND16G_00003057), myo (ZIND16G_00001094), robo2 (ZIND16G_00008188), loco (ZIND16G_00001880), wit (ZIND16G_00000511), CG33958 (ZIND16G_00004664), Duox (ZIND16G_00005661), numb (ZIND16G_00007698)
**Cell movements and encapsulation of foreign target** ***Upregulated*:** Strn-Mlck (ZIND16G_00004905), hh (ZIND16G_00003155), Pvf3 (ZIND16G_00008540), Itgbn (ZIND16G_00009296), p130CAS (ZIND16G_00006792), if (ZIND16G_00005558), Dhc16F (ZIND16G_00008232), AdamTS-A (ZIND16G_00003414), trio (ZIND16G_00001152), cher (ZIND16G_00004253), CycB (ZIND16G_00004885), unc-104 (ZIND16G_00004506), ncd (ZIND16G_00005467), mew (ZIND16G_00004434), scb (ZIND16G_00007892), trbd (ZIND16G_00008915), Rala (ZIND16G_00002638), Nrg (ZIND16G_00002077), Eb1 (ZIND16G_00006903) ***Downregulated*:** m (ZIND16G_00004819), unc-5 (ZIND16G_00007900), ena (ZIND16G_00003069), Invadolysin (ZIND16G_00005474), Lar (ZIND16G_00009598), robo2 (ZIND16G_00008188), stai (ZIND16G_00001760)
**Production of energy source** ***Upregulated*:** CG5171 (ZIND16G_00007239), Treh (ZIND16G_00009882), Men-b (ZIND16G_00000144), beta-Man (ZIND16G_00007729), Ldh (ZIND16G_00008314), Gnmt (ZIND16G_00004579) ***Downregulated:*** Oscillin (ZIND16G_00006234), Pdk (ZIND16G_00009519), Gfat1 (ZIND16G_00003438), fbp (ZIND16G_00009604), foxo (ZIND16G_00008428)
**Melanization** ***Upregulated*:** Sp7 (ZIND16G_00001050) ***Downregulated:*** modSP (ZIND16G_00002867), PGRP-LC (ZIND16G_00008135)
**Elimination of microorganisms** ***Upregulated*:** grass (ZIND16G_00000143), PGRP-SB1 (ZIND16G_00000431), CecC (ZIND16G_00002978), DptB (ZIND16G_00002658), AttD (ZIND16G_00001258), Sp7 (ZIND16G_00001050), Tep2 (ZIND16G_00008527), IM33 (ZIND16G_00009971), Bbd (ZIND16G_00006553), slif (ZIND16G_00005220), CG16799 (ZIND16G_00006321), Tep3 (ZIND16G_00009332), PGRP-SC2 (ZIND16G_00004509), PGRP-LB (ZIND16G_00003503), Der-2 (ZIND16G_00003566), Rala (ZIND16G_00002638) ***Downregulated:*** PGRP-LC (ZIND16G_00008135), modSP (ZIND16G_00002867), Tab2 (ZIND16G_00007372), CG13551 (ZIND16G_00005409), spz (ZIND16G_00008556), NimC1 (ZIND16G_00002951), Duox (ZIND16G_00005661)

Second, because the derivatives of the giant hemocytes, the anucleated fragments, detected at the wound sites are present in both the uninduced and induced samples, we searched for constitutively expressed genes that could potentially be involved in wound healing processes. On the one hand, we selected those genes that had *D. melanogaster* orthologs known to be involved in blood coagulation, wound healing, and cuticle remediation (**Table 2**). On the other hand, we selected those constitutively expressed *Z. indianus* genes that had mammalian orthologs involved in the function of megakaryocytes and platelets (**Supplementary Table 3**).

**Table 2:** Set of constitutively expressed *Zaprionus indianus* genes encoding for orthologs of *Drosophila melanogaster* proteins involved in wound healing and hemolymph clotting.

Z. indianus gene ID	D. melanogaster ortholog	Role in D. melanogaster
ZIND16G_00008476	fon	Fat body-secreted hemolymph clotting factor.
ZIND16G_00008262	Hml	Hemolymph clotting.
ZIND16G_00006641	PPO2	Melanization of wounds and capsules.
ZIND16G_00006889	PPO1	Melanization of wounds.
ZIND16G_00008507	CG42259	Involved in response to wounding.
ZIND16G_00003920	Glt	Cross-links blood clot.
ZIND16G_00001938	pbl	Wound healing.
ZIND16G_00006474	Tg	Cuticle development. Hemolymph coagulation, wound healing.
ZIND16G_00002297	Cht2	Cuticle development. Wound healing.
ZIND16G_00006825	Fhos	Plasmatocyte migration during immune response, autophagic programmed cell death.
ZIND16G_00007154	CG15170	Wound healing.
ZIND16G_00008356	TTLL4A	Wound healing.
ZIND16G_00007461	Mtl	Dorsal closure, wound healing, cell migration.
ZIND16G_00005073	Coq3	Wound healing.
ZIND16G_00008371	holn1	Wound healing.
ZIND16G_00004207	CG11089	Wound repair.
ZIND16G_00000950	CG6005	Wound healing.
ZIND16G_00009846	Mmp2	Cleaves proteins in the extracellular matrix. Wound healing.
ZIND16G_00001627	Idgf3	Component of hemolymph clot.
ZIND16G_00006536	CG3294	Splicing factor involved in wound healing.
ZIND16G_00001159	Cht7	Chitin-based cuticle development.
ZIND16G_00003151	Hmu	Wound healing.

As mentioned above, three of the expanded and rapidly evolving gene families, expressed either differentially or constitutively in the blood cells, are of special interest: the serine proteases, the FREPs, and the C-type lectins. The serine proteases (24–26) comprise a highly expanded class, and some of them act in complement-like cascades to activate Toll signaling, melanization, or coagulation. The FREPs (27–30) and the C-type lectins (31, 32) are pathogen-binding proteins that play important roles in the immune defense of many animals. Of the 90 expressed *Z. indianus* serine proteases, 54 have specific *D. melanogaster* orthologs (**Supplementary Table 4**). The identified orthologs include genes with known roles in immunity, such as *Hayan*, *Sp7* in PPO1/2 activation, and *grass* and *modSP* in Toll signaling. The remaining 36 genes belong to *Z. indianus*-specific clades, similar to the immunity-related serine proteases, but lack obvious 1:1 orthology relationships with *D. melanogaster* homologs. Of the 51 FREP genes, only 22 have *D. melanogaster* orthologs, and most of these genes have not been characterized so far (**Supplementary Table 4**). The situation is similar for the 18 *Z. indianus* lectin genes, as only five have *D. melanogaster* orthologs (**Supplementary Table 4**). The differentially expressed serine proteases, FREPs, and C-type lectins could be involved in the efficient parasitoid-killing processes of *Z. indianus* (**Supplementary Table 4**).

Furthermore, regarding signaling pathways involved in blood cell differentiation and function, we found components of the JNK pathway (Alg-2, bsk, CYLD, Cdc37, jra, msn, and Pvr) and elements of the JAK–STAT pathway (hop, Stat92E, dome, Ptp61F, and Socs36E) constitutively expressed in blood cells isolated from both naïve and induced larvae (**Supplementary Table 1**). However, upd1 and upd3 genes, which encode the ligands of the JAK–STAT signaling, had no or very low expression, and the upd2 ortholog is not encoded in the genome of *Z. indianus* (21).

### 3.6 Comparison to wasp-induced genes in *D. melanogaster* and *D. ananassae* hemocytes

Although the morphological features of lamellocytes in wasp-infected *D. melanogaster* larvae are very different from those of the MGHs and large plasmatocytes in *Z. indianus*, we were interested in whether the genes that were upregulated in *Z. indianus* include orthologs of lamellocyte-specific genes of *D. melanogaster*. Genes that are specific to different *D. melanogaster* hemocyte classes have recently been identified in six independent single-cell sequencing projects (33–38), and consensus lists of these data have been compiled (9). *Z. indianus* homologs of 21 *D. melanogaster* lamellocyte marker genes were indeed upregulated in hemocytes after wasp infection, while three were downregulated (**Supplementary Table 5**). Thus, there seems to be some correlation between the activity of activated *Z. indianus* hemocytes and *D. melanogaster* lamellocytes. The upregulated genes include orthologs of classical lamellocyte markers such as *atilla*, *cher*, and the integrins *Itgbn* and *mew*, as well as several genes involved in cytoskeletal organization and sugar import.

In contrast to the lamellocyte markers, many plasmatocyte and crystal cell marker homologs were downregulated in *Z. indianus* hemocytes after wasp infection (**Supplementary Table 5**), albeit modestly so. This may reflect a decreased relative proportion of the corresponding cell classes in the infected animal.

We also compared the differentially expressed genes in *D. ananassae* MGHs and activated plasmatocytes (12) with the differentially expressed *Z. indianus* genes. We noted that expression of six of the lamellocyte markers, *atilla*, *Itgbn*, *Trehalase*, the sugar transporter *CG1208*, *Esyt2*, and Gdap2, were highly expressed in wasp-infected blood cells of *Z. indianus* and also showed increased expression in MGHs of *D. ananassae*, while three of the highly expressed *Z. indianus* genes, *Trehalase*, *Esyt2*, and *aru*, were also upregulated in the activated plasmatocytes of *D. ananassae* (**Supplementary Table 6**). However, at present, it is hard to say if this overlap in gene expression in effector hemocytes from these three species is due to convergent evolution or if it reflects a common origin of the involved cell types, despite their very different appearances.

## 4 Discussion

Drosophilids have developed different strategies to circumvent parasitoid attack, which usually involves the transdifferentiation of phagocytic plasmatocytes to non-phagocytic, encapsulating cell types. Several specialized cell types have been described as lamellocytes in *D. melanogaster* (3), MGHs in *Z. indianus* (11), and species of the *ananassae* subgroup (10), giant hemocytes, and nematocytes in *Z. indianus* (11, 19) that encapsulate parasitoids. However, it is not yet known how they are related to each other. When the hosts co-evolve with their local parasite communities, they may acquire novel elements in the arms race, e.g., different types of effector cells develop against the co-evolving parasitoid. This study was carried out with the aim to test the specific features of defense in an invasive *Drosophila* species, *Z. indianus*, which develops different types of multinucleated cells in response to parasitoid attack.

We found that *D. ananassae* and *Z. indianus*, using MGHs in the defense against parasitoid wasps, generated highly effective innate immune responses when compared to *D. melanogaster*, which uses lamellocytes to protect against the invaders (**Figure 1**). The presence of multinucleated hemocytes among Drosophilidae, apart from species of the *ananassae* subgroup (10), and *Z. indianus* (11) was also detected in *Drosophila falleni* and *Drosophila phalerata* (39). The broad distribution of multinucleated hemocytes within the family suggests that this cell type could be present in a common ancestor of Drosophilidae, and the lamellocytes in the *melanogaster* subgroup possibly have turned up as a novelty, where they have replaced the MGHs (9). Previously, we observed that MGHs, a syncytium of blood cells with several nuclei in *D. ananassae* and *Z. indianus*, could develop by fusion of elongated hemocytes (10, 11). The specific features of this cell type may underlie the effective immune response of these species.

A novel cell type, the spherical large plasmatocyte with characteristic features of both the plasmatocytes and the giant cells (including MGHs and nematocytes) as well as the anucleated cytoplasmic fragments, could be one key component of the immune response of *Z. indianus*. Large plasmatocytes develop specifically after parasitoid wasp infection. Bacteria, fungi, or inert foreign particles do not cause differentiation of large plasmatocytes (**Figure 3**); hence, we hypothesize that components of the egg, the venom, and virus-like particles injected during oviposition may be recognized by the pattern recognition factors of the host and may serve as special triggers and promote differentiation of these cells. Large plasmatocytes could be key components of the anti-parasitoid response, producing substances targeted against the invaders, or they may serve as an intermediate cell subpopulation through MGH differentiation. Large plasmatocytes carry one, two, or more nuclei of different sizes and occasionally express the 4G7 antigen, a characteristic feature of giant hemocytes (**Figure 4**) (11), and their phagocytic capacity is lower than that of the normal-sized plasmatocytes (**Figure 5**). Video microscopy showed that large, spherical cells, most probably large plasmatocytes, rapidly change shape and become elongated, vigorously moving cells (**Supplementary Movie 1**), implying that they apparently differentiate into giant hemocytes. The elongated shape and high motility of the giant hemocytes could provide highly effective encapsulation capacity for this cell type.

Though the 4G7-positive, encapsulating cell fraction, including MGHs, nematocytes, and anucleated cytoplasmic fragments, increased in number and size after parasitoid infection, this subpopulation was also present in naïve *Z. indianus* larvae. Here, we provide evidence for a novel function of the giant hemocytes, as we found that in both naïve and wasp-infected larvae, they were present at wound sites and hence could be involved in the wound healing process. This finding is also supported by the ultrastructural similarities observed between the giant hemocytes of *Z. indianus* and the mammalian megakaryocytes. Both cell types have an elaborated open canalicular system communicating with the extracellular space, and both release a large number of anucleated cell fragments (11, 40). Anucleated cell fragments were also described in *D. falleni* and *D. phalerata*, where they carry mRNA to translate into proteins (39), which provides them remarkable autonomy, similar to mammalian platelets (41, 42).

*D. melanogaster* possesses the wound healing machinery, which involves hemolymph components secreted by the fat body, such as fondue and glutacin, and hemocyte-derived factors, such as hemolectin, hemomucin, transglutaminase, and phenoloxidases, which are all involved in the formation of a fibrous or gelatinous network at the epithelial breaches, thus sealing the damaged tissue (43–45). Interestingly, multinucleated epidermal cells were also detected at the *Drosophila* wound sites, which helped to eliminate the intracellular spaces and restore tissue integrity (18). Transcriptomic analysis of *Z. indianus* blood cells, on the basis of the analysis of their *D. melanogaster* orthologs, revealed constitutive expression of several genes, which could be involved in wound healing and coagulation processes (**Table 2**). Furthermore, we identified several genes expressed in *Z. indianus* blood cells, which encoded orthologs of proteins involved in mammalian blood coagulation (**Supplementary Table 3**); hence, they may also be involved in wound healing in this insect species. Based on the involvement of *Z. indianus* anucleated fragments in wound healing processes, here, we present an example where, in phylogenetically distant species, small, anucleated cell fragments with special ultrastructure evolved through distinct evolutionary trajectories in insects and mammals to act in similar biological processes.

Transcriptomic analysis revealed further characteristic features of gene expression patterns in this species. Several differentially expressed genes in naïve and wasp-induced hemocyte samples could be involved in the immune response against parasitoid wasps (**Supplementary Table 2**). Based on previous studies (33–38), *D. melanogaster* genes, such as *atilla*, *itgbn*, *Treh*, Esyt2, and CG1208, were enriched in the lamellocytes and were also expressed at significantly higher levels in the MGHs of *D. ananassae* and the blood cells of parasitoid-infected *Z. indianus*; hence, they could be involved in the anti-parasitoid defense of each species (**Supplementary Table 5**, **Supplementary Table 6**). We have also shown rapidly evolving gene families to be expressed in the transcriptome of *Z. indianus* blood cells, including FREPs and C-type lectins, which have been described to act as pattern recognition factors in other insect species. Hence, in *Z. indianus*, blood cell differentiation may be induced through them.

The JAK–STAT signaling pathway has been highly conserved throughout evolution, as it is involved in the regulation of immune responses in mammals (46) and also controls different steps of hematopoiesis and the response to immune challenge in *D. melanogaster* (47). We previously found that gene orthologs encoding elements of this pathway were not enriched in MGHs of *D. ananassae* (12). Here, we have shown that, although some components of the JAK–STAT pathway were expressed constitutively, upd1 and upd3 genes, encoding the ligands of this pathway, had no or very low expression, while the upd2 ortholog is not encoded by the genome of *Z. indianus* (21). However, several components of the JNK pathway, including bsk and Cdc37 genes involved in the activation of the pathway (48, 49), were expressed at a higher level in blood cells isolated from wasp-infected *Z. indianus* samples (**Supplementary Table 1**), indicating that JNK signaling is likely involved in blood cell differentiation after parasitoid infection.

Finally, we conclude that the blood cells of *Z. indianus* generally represent an extremely plastic population of cells, which could contribute to the highly efficient immune response against parasitoids and may also participate in defense processes such as wound healing and cuticle remediation. Our research provides insights into the differentiation and function of blood cells, highlights the importance of different molecules involved in blood cell-mediated responses, and suggests possible model organisms for further investigations.

## Data availability statement

The data presented in the study are deposited in the NCBI SRA repository, accession number PRJNA1041101.

## Ethics statement

The manuscript presents research on animals that does not require ethical approval for their study.

## Author contributions

GC: Conceptualization, Data curation, Funding acquisition, Investigation, Methodology, Project administration, Supervision, Validation, Visualization, Writing – original draft, Writing – review & editing. LM: Conceptualization, Data curation, Formal Analysis, Funding acquisition, Investigation, Methodology, Software, Validation, Visualization, Writing – original draft, Writing – review & editing. HK: Data curation, Methodology, Validation, Visualization, Writing – original draft. VV: Data curation, Validation, Visualization, Writing – original draft. LB: Data curation, Investigation, Resources, Software, Writing – original draft. NZ: Data curation, Investigation, Software, Writing – original draft. GN: Data curation, Software, Writing – original draft. ZH: Data curation, Software, Writing – original draft. DH: Conceptualization, Data curation, Formal Analysis, Funding acquisition, Software, Validation, Writing – original draft, Writing – review & editing. IA: Conceptualization, Funding acquisition, Investigation, Methodology, Project administration, Supervision, Validation, Visualization, Writing – original draft, Writing – review & editing.

## Supplementary material

Supplementary Figure 1 The wound site of an uninduced *Z. indianus* larva. The image corresponds to Supplementary Movie 2, and was captured using an Olympus FV1000 confocal LSM microscope, 2 h after sterile wounding.

Supplementary Figure 2 GO enrichment analysis in the “biological process” category for the differentially expressed genes between the induced and uninduced samples. Genes that were significantly upregulated in the induced samples are labeled in green and those that were significantly downregulated are labeled in red. GO terms are plotted according to the significance of their enrichment (-log10 p-value).

Supplementary Figure 3 GO enrichment analysis in the “cellular component” category for the differentially expressed genes between the induced and uninduced samples. Genes that were significantly upregulated in the induced samples are labeled in green and those that were significantly downregulated are labeled in red. GO terms are plotted according to the significance of their enrichment (-log10 p-value).

Supplementary Table 1 List of the *Z. indianus* genes expressed in the blood cells of uninduced and *L. victoriae*-infected larvae. FPKM values show gene expression levels for each sample. *D. melanogaster* and *D. ananassae* orthologs are listed.

Supplementary Table 2 Set of genes differentially expressed in uninduced and *L. victoriae*-induced *Z. indianus* blood cells. Positive and negative log2FoldChange values indicate higher and lower gene expression levels, respectively, in induced compared to uninduced samples. Genes expressed exclusively in uninduced samples are labeled with a light grey background and those expressed exclusively in induced samples are labeled with a dark grey background. *D. melanogaster* and *D. ananassae* orthologs are listed.

Supplementary Table 3 List of the mammalian coagulation-associated genes possessing constitutively expressed orthologs in *Z. indianus* blood cells. The corresponding *D. melanogaster* and *D. ananassae* orthologs are also shown.

Supplementary Table 4 Set of putative serine protease, FREPs, and C-type lectin genes expressed either differentially (grey) or constitutively in *Z. indianus* blood cells. *D. melanogaster* and *D. ananassae* orthologs are listed. Not applicable (NA).

Supplementary Table 5
*D. melanogaster* blood cell specific markers expressed differentially in *Z. indianus*. Data originating from single cell RNA sequencing of lamellocytes (LC), crystal cells (CC), and plasmatocytes (PC).

Supplementary Table 6 Common differentially expressed genes in the blood cells of *Z. indianus* and *D. ananassae*. Activated plamsatocytes (aPC), naïve hemocytes (nHC).

Supplementary Movie 1 A large plasmatocyte differentiating into an elongated giant hemocyte. Blood cells were isolated 48 h following *L. victoriae* parasitoid wasp infection. Shooting duration: 75 min.

Supplementary Movie 2 Three-dimensional confocal picture reconstruction of a 4G7- and DAPI-stained wound. Wounding was generated using a 100 μm diameter sterile minutien pin on an uninduced larva. Analysis was performed 2 h after wounding using an Olympus FV1000 confocal LSM microscope. The corresponding trans light image is presented in Supplementary Figure 1.
