# Supplementary figures and images for: Distinctive features of Zaprionus indianus hemocyte differentiation and function revealed by transcriptomic analysis
Source: Front Immunol. 2023 Dec 21;14:1322381. doi: 10.3389/fimmu.2023.1322381 (PMC10768004; doi:10.3389/fimmu.2023.1322381)

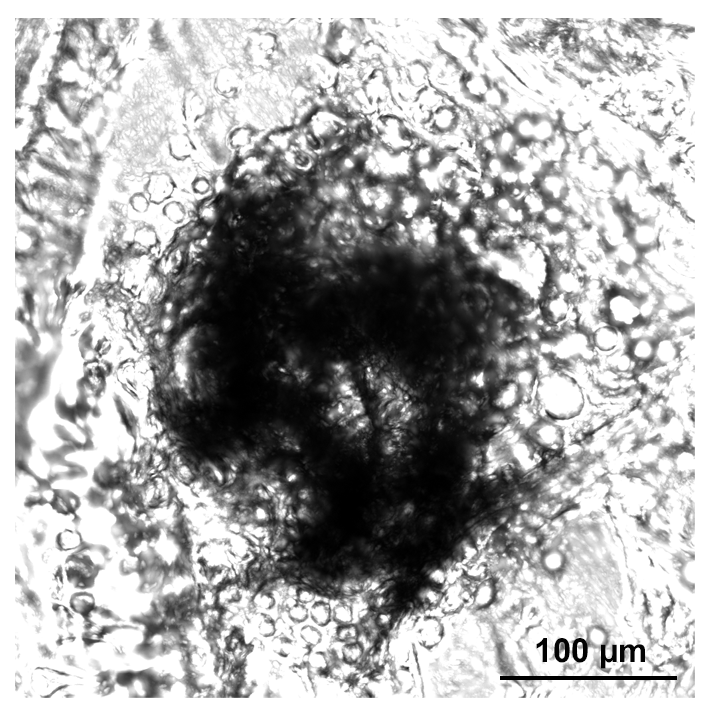

Supplement: Supplementary Figure 1 — The wound site of an uninduced Z. indianus larva. The image corresponds to Supplementary Movie 2, and was captured using an Olympus FV1000 confocal LSM microscope, 2 h after sterile wounding. [file Image_1.tif]

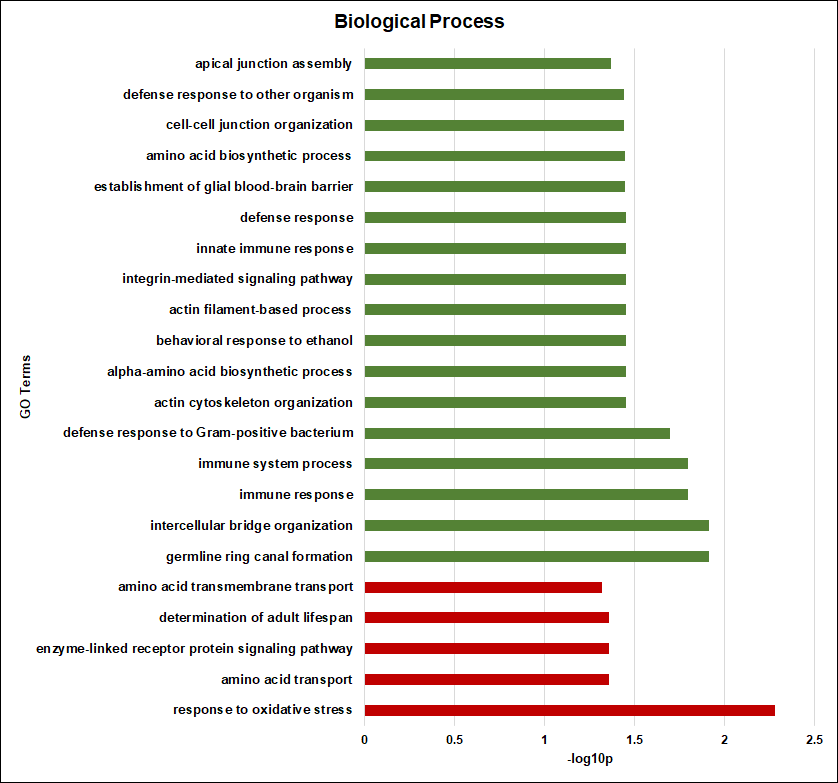

Supplement: Supplementary Figure 2 — GO enrichment analysis in the “biological process” category for the differentially expressed genes between the induced and uninduced samples. Genes that were significantly upregulated in the induced samples are labeled in green and those that were significantly downregulated are labeled in red. GO terms are plotted according to the significance of their enrichment (-log10 p-value). [file Image_2.tif]

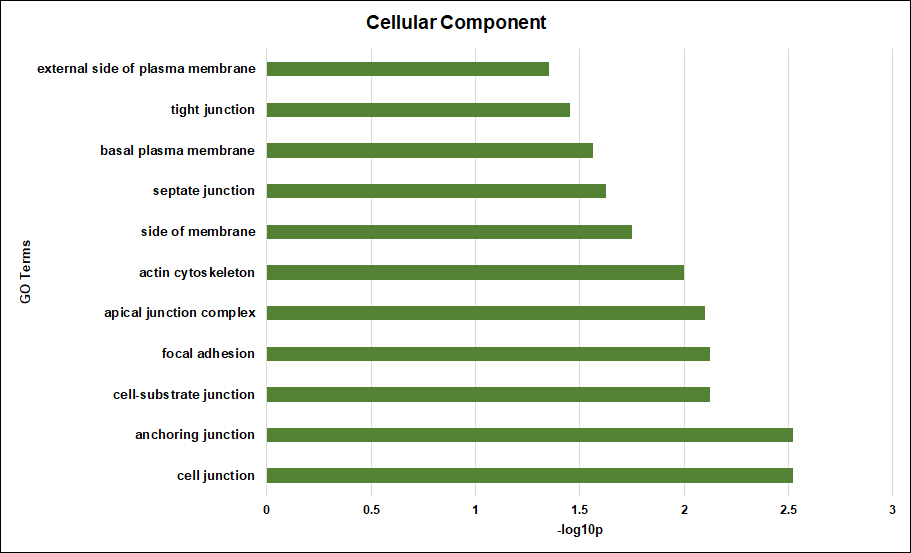

Supplement: Supplementary Figure 3 — GO enrichment analysis in the “cellular component” category for the differentially expressed genes between the induced and uninduced samples. Genes that were significantly upregulated in the induced samples are labeled in green and those that were significantly downregulated are labeled in red. GO terms are plotted according to the significance of their enrichment (-log10 p-value). [file Image_3.tif]
